# Association of Patient and Site-of-Care Characteristics With Reproductive Carrier Screening Timing in a Large Integrated Health System

**DOI:** 10.1001/jamanetworkopen.2022.40829

**Published:** 2022-11-08

**Authors:** Leland E. Hull, David Cheng, Mie H. Hallman, M. L. Rieu-Werden, Jennifer S. Haas

**Affiliations:** 1 Division of General Internal Medicine, Massachusetts General Hospital, Boston; 2 Harvard Medical School, Boston, Massachusetts; 3 Biostatistics Center, Massachusetts General Hospital, Boston, Massachusetts

## Abstract

**Importance:**

Reproductive genetic carrier screening can be performed prior to or during pregnancy to assess a reproductive couple’s risk of having a child with a recessively inherited disorder. Although professional societies endorse preconception screening as preferable to prenatal screening to allow for greater reproductive choice, implementation of preconception screening is challenging.

**Objective:**

To determine how carrier screening timing varies by multilevel factors associated with health care delivery including patient, clinician, and location across a large integrated health care system.

**Design, Setting, and Participants:**

This cross-sectional study used a mixed-methods approach including (1) quantitative analysis of multilevel factors associated with the timing of reproductive carrier screening and (2) qualitative analyses of data from interviews conducted with clinicians ordering carrier screenings. The setting was the Mass General Brigham, a large integrated health care system in the greater Boston, Massachusetts area. Participants included adult female patients who completed reproductive carrier screening performed by Myriad Women’s Health between October 1, 2018, to September 30, 2019.

**Exposures:**

Site of care (ordering clinical location and hospital affiliate), ordering clinician specialty, and patient characteristics, including age at date of test collection, self-reported race and ethnicity, primary insurance payor, and number of comorbidities.

**Main Outcomes and Measures:**

The primary outcome was the timing of carrier screening (preconception vs prenatal). A series of 4 multilevel logistic regression models were fitted to measure the relative contribution of site, clinician, and patient-level factors on the timing of screening. Interviews with ordering clinicians (N = 9) were analyzed using a framework approach to explore barriers to preconception screening.

**Results:**

Among 6509 adult female patients who completed carrier screenings, 770 (12%) were Asian, 352 (5%) were Hispanic, 640 (10%) were non-Hispanic Black, 3844 (59%) were non-Hispanic White, 858 (13%) were other or multiple races and ethnicities, and 2611 (40%) were aged 31 to 35 years; 4701 (63%) had prenatal screening and 2438 (37%) had preconception screening; screenings were ordered by 161 distinct clinicians across 32 clinical locations affiliated with 4 hospitals. In model 1, adjusted for hospital (fixed effect), clinic and clinician (random effects), 49% of the variability in timing was associated with clinician-level effects (intraclass correlation coefficient [ICC], 0.49) and 28% was associated with clinic-level effects (ICC, 0.28). Clinician specialty explained the greatest amount of variation in screening timing. Interviewed clinicians (N = 9) supported preconception screening but cited several barriers to offering population-based preconception screening.

**Conclusions and Relevance:**

In this cross-sectional study, multilevel factors were associated with carrier screening timing. These findings suggest that increasing access to preconception screening may involve engaging specific medical specialties.

## Key Points

**Question:**

Are site of care, clinician, and patient-specific factors associated with differences in whether reproductive genetic carrier screening was ordered prior to vs during pregnancy across a health care system?

**Findings:**

This cross-sectional study of 6509 patients who completed carrier screening ordered across the Mass General Brigham integrated health care system in Boston, Massachusetts, including 4 hospitals, 32 clinical sites, and 161 ordering clinicians, from October 1, 2018, through September 30, 2019, found that most (63%) had prenatal screening. Clinician specialty was associated with the greatest variation in timing among observed characteristics.

**Meaning:**

These findings suggest that engaging clinicians who see patients prior to pregnancy may improve access to preconception carrier screening.

## Teaser

This cross-sectional study examines how carrier screening timing varies by multilevel factors associated with health care delivery, including patient, clinician, and location across a large integrated health care system.

## Introduction

Reproductive genetic carrier screening can be performed prior to or during pregnancy to inform a reproductive couple’s risk of having a child affected by an X-linked or autosomal recessive inherited disease. Carriers may not exhibit overt clinical symptoms, but their children may be at risk of the disease.^1^ Identifying at-risk parents allows them to seek genetic counseling and can inform reproductive decision-making. Population-based carrier screening programs have been implemented for several severe recessive conditions, such as Tay-Sachs disease and cystic fibrosis.^2,3^ Technological advances have increased our ability to identify couples at risk for having a child affected by a severe recessive disease,^4,5^ many times in a cost-effective manner.^6^

Both the American College of Obstetricians and Gynecologists (ACOG) and the American College of Medical Genetics recommend that carrier screening be offered to patients during, or preferably, prior to pregnancy to allow prospective parents greater reproductive choice.^7,8,9^ Couples who are identified as being at risk for having a child affected by severe recessive disease preconception have greater time to process this information, incorporate this information in their reproductive planning, and, in some cases, consider the use of advanced reproductive technologies to prevent having a child affected by the genetic disease. The recent overruling of Roe vs Wade could also limit the ability of reproductive couples to make use of the information provided by prenatal screening.^10^

However, implementation of preconception screening is complicated. It requires identifying patients prior to conception, coordinating their testing and possibly that of a reproductive partner, delivering the results, and incorporating these results into patient care.^11,12^ It is well established that factors at multiple levels of health care affect the quality and delivery of health interventions.^13^ Therefore, we sought to quantify the extent to which variability in timing of screening (preconception vs prenatal) was explained by site-, clinician-, and patient-level factors across a large integrated health care system and explore potential explanations for the observed variability.

## Methods

### Overview

We conducted a cross-sectional study using a mixed-methods approach consisting of (1) quantitative analyses of the hospital-, clinic-, clinician-, and patient-level factors associated with the timing of reproductive carrier screening and (2) qualitative data from explanatory interviews conducted with a subset of clinicians ordering carrier screens. This study followed the Strengthening the Reporting of Observational Studies in Epidemiology (STROBE) reporting guideline for cross-sectional studies^14^ and Consolidated Criteria for Reporting Qualitative Research (COREQ) reporting guideline^15^ checklist for reporting qualitative data. Study procedures were deemed exempt by the Mass General Brigham (MGB) institutional review board because this study posed minimal risk. Interviewed clinicians consented verbally.

### Data Sources and Study Population

The MGB HealthCare Network^16^ is an integrated health care system that provides care for approximately half of the population in the greater Boston region. The system includes Brigham and Women’s Hospital (BWH), BWH’s Faulkner Hospital, Newton-Wellesley Hospital (NWH), Massachusetts General Hospital (MGH), and Salem Hospital (called North Shore Medical Center [NSMC] at the time of this study).^16^ Ambulatory patients are seen at clinics, practices, and community health centers affiliated with these different MGB hospitals. During the study period, more than 32 000 adult female sex-at-birth patients were seen for pregnancy-related care (*International Statistical Classification of Diseases and Related Health Problems, Tenth Revision [ICD-10]* diagnosis code of 000-O9A); most self-identified as White race (64%) and non-Hispanic ethnicity (84%), and relatively few had Medicaid and Medicare claims data available (8%).^16^

Our study population included adult female individuals of reproductive age (18 to 51 years) who completed carrier screening through Myriad Genetics Inc, the predominant laboratory used for carrier screening across MGB, during fiscal year (FY) 2019 (October 1, 2018, through September 30, 2019). We enacted a data use agreement with Myriad Genetics Inc, to obtain the following data for patients who completed carrier screening through their laboratory, including patient age at testing, dates of test collection, the ordering clinician’s name and National Provider Identifier (NPI) number, the address affiliated with the test order, and the hospital affiliated with the ordering address, and test characteristics (number of conditions tested for and test results). These data were linked to electronic health record (EHR) data from both MGB’s Research Patient Data Registry (RPDR)^16,17^ and the MGB Enterprise Data Warehouse, including patient demographic files, encounter files, *ICD-10* diagnosis codes, obstetrical radiological imaging, and pregnancy episode dates to characterize the patients undergoing testing and ascertain their pregnancy status at the time of testing.

Only patients who had carrier screening completed via Myriad Genetics Inc and whose test records were able to be linked to EHR data (eFigure 1, eTable 1 in the Supplement) were retained in the final cohort. For the small proportion of patients with more than 1 carrier screening performed during FY 2019 (n = 84; less than 2% of cohort), we retained the first carrier screening ordered. Most of these patients (61 of 84) had the same pregnancy status for both screenings.

### Outcome Classification

The primary outcome was reproductive carrier screening timing (preconception vs prenatal). A custom algorithm was developed to ascertain pregnancy status at the time of carrier screening. A screening was considered to have been ordered prenatal if it was ordered within 4 weeks of a screening obstetric radiology examination or within 2 weeks of an EHR indicator of pregnancy start date. Tests not meeting these criteria were classified as being performed preconception. Manual medical record review of 100 random patient medical records demonstrated greater than 96% sensitivity and 100% specificity of the algorithm for estimating pregnancy status accurately.

### Covariates

Patient-level data included the patient’s age at date of test collection or, if unavailable, when received at the laboratory, self-reported race and ethnicity from the EHR (Asian, Non-Hispanic Black, Hispanic, Other [at least 2 selections, American Indian or Alaska Native, Hawaiian, Pacific Islander), Non-Hispanic White, Unknown [missing/prefer not to answer]),^18^ primary insurance payor listed for the clinical encounter closest to the date when screening was ordered (Public [Medicaid or Medicare], None, Private), and number of comorbidities.^19^ Clinician-level data included an indicator for the ordering clinician of record for each test, and the ordering clinician’s specialty, based on manual review of MGB webpage profiles (General Obstetrician-Gynecologists [OB/Gyn], Maternal Fetal Medicine [MFM], Reproductive Endocrinology/Infertility [REI], Other medical specialties, certified nurse midwives [CNM], nurse practitioner [NP]/physician’s assistants [PA]). Nurse midwives were analyzed separately from NPs/PAs given their specialization in pregnancy care. Site-level data included data from 2 sublevels. First, an indicator of the ordering clinical location was assigned based on the 32 distinct test order addresses. The second level of site data included the hospital affiliate for each clinic site (BWH, MGH, NSMC, and NWH). Faulkner Hospital, a single clinical site, was included as a distinct BWH site. Test characteristics were available but not used in these analyses given the strong association between test timing and testing characteristics (eFigure 3 in the Supplement); these may be informative for future assessments of clinical outcomes associated with carrier screening.

### Statistical Analyses

Patient and clinician-level characteristics associated with screening timing were summarized by timing using χ^2^ or Fisher exact tests for comparison between groups dependent on cell sizes. To account for clustering withing clinicians and sites of care, we fit a series of multilevel logistic regression models to assess the variability in carrier screening timing at the patient, clinician, clinic, and hospital levels and to identify factors associated with preconception screening.^20,21^An initial model including fixed effects for hospital and random intercepts for distinct clinicians and clinics (model 1) was fit to assess variability at each level without adjustment. We then fit models that additionally included either fixed effects for the clinician specialty (model 2) or for patient level characteristics (model 3) to assess how much observed factors at different levels independently explain variation in timing. Finally, we fit a model including fixed effects for both specialty and patient-level characteristics (model 4) to estimate adjusted associations.

The variability at the clinician, clinic, and hospital levels in each model was characterized by a suite of metrics.^20,22^ The SD is a measure of residual variability across the associated level after adjusting for fixed effects. The proportion change in variance (PCV) measures how this variability changes relative to model 1 after adjusting for patient- and clinician-level characteristics. The intraclass correlation coefficient, (ICC), calculated under a latent response formulation, quantifies the share of total variability attributable to the clinician and clinic levels on a scale of 0-1.^23^ The median odds ratio (MOR) estimates the median of odds ratios that would result from sampling patients higher and lower risk of being screened preconception, chosen from within the same clinician or clinic and with the same covariates.^22^ The heterogeneity across clinicians and sites was also evaluated by plotting the predicted probability of preconception screening and corresponding prediction intervals across clinicians by hospital and clinics under model 1.^24^

Two-sided *P*. < 05 were interpreted as statistically significant. SAS version 9.4 (SAS Institute Inc) was used for cohort building and cleaning; statistical analyses were conducted using R version 4.0.2 (R Project for Statistical Computing). Analyses were performed from February to September 2022.

#### Explanatory Interviews

To better understand variability in carrier screening, we conducted semistructured telephone interviews of ordering clinicians who demonstrated variability in carrier screening ordering practices during the study period. We used the Consolidated Framework for Implementation Research (CFIR),^25,26^ to direct interview guide design and a rapid qualitative matrix approach for data analysis.^27,28^ Results were organized by CFIR domain. Details of qualitative methods are available in the eAppendix of the Supplement.

## Results

### Cohort Characteristics

Our final study cohort included 6509 adult female individuals, of whom 770 (12%) were Asian, 352 (5%) were Hispanic, 640 (10%) were non-Hispanic Black, 3844 (59%) were non-Hispanic White, 858 (13%) were other or multiple races and ethnicities, and 2611 (40%) were aged 31 to 35 years; 4701 (63%) had prenatal screening and 2438 (37%) had preconception screening (Table 1). Patients screened preconception were older and more frequently self-reported White Non-Hispanic race compared with those screened prenatally; less had public insurance. General OB/Gyns (n = 70) ordered the most carrier screens of any clinician specialty (n = 2614, 40% of total), whereas REIs ordered the most preconception testing (n = 1795). A handful of family medicine and internal medicine physicians (n = 7 clinicians), clinical genetics specialists (n = 3 clinicians), and a single pulmonologist ordered exclusively preconception screens. BWH and MGH accounted for most of the carrier screens ordered across the health care system.

**Table 1.** Characteristics of Patients, Ordering Clinicians, and Order Location by Carrier Screening Timing

Characteristic	Total screenings, No. (% of total) (N = **6509**)	Preconception screenings, No. (% within group) (n = 2438)	Prenatal screenings, No. (% within group) (n = 4071)	*P* value
Patient age, y				
18-25	597 (9)	75 (13)	522 (87)	<.001
26-30	1743 (27)	534 (31)	1209 (69)	
31-35	2611 (40)	1012 (39)	1599 (61)	
36-40	1283 (20)	648 (51)	635 (49)	
≥41	275 (4)	169 (61)	106 (39)	
Patient race and ethnicity				
Asian	770 (12)	304 (39)	466 (61)	<.001
Black non-Hispanic	640 (10)	176 (28)	464 (73)	
Hispanic	352 (5)	80 (23)	272 (77)	
White non-Hispanic	3844 (59)	1656 (43)	2188 (57)	
Other/multiple race	858 (13)	190 (22)	668 (78)	
Unknown/missing	45 (1)	32 (71)	13 (29)	
Patient insurance^a^				
Public	1379 (21)	195 (14)	1184 (86)	<.001
Private	3436 (53)	1551 (45)	1885 (55)	
None	1618 (25)	663 (41)	955 (59)	
Patient comorbidities^a^				
0 comorbidity	5074 (78)	1921 (38)	3153 (62)	<.001
1 comorbidity	1196 (18)	396 (33)	800 (67)	
2 or more	219 (3)	102 (47)	117 (53)	
Clinician specialty (clinicians, total n = 161)^b^				
OB/Gyn (n = 70)	2614 (40)	269 (10)	2345 (90)	<.001
REI (n = 21)	1847 (28)	1795 (97)	52 (3)	
MFM (n = 26)	789 (13)	246 (31)	543 (69)	
Midwife (n = 22)	703 (11)	7 (1)	696 (99)	
PA/NP (n = 11)	500 (8)	65 (13)	435 (87)	
Other (n = 11)^c^	56 (1)	56 (100)	0 (0)	
Hospital (clinics, total n = 32)^d^				
BWH/Faulkner (n = 17)	2756 (42)	1197 (43)	1559 (57)	<.001
MGH (n = 10)	3064 (47)	1067 (35)	1997 (65)	
NWH (n = 1)	174 (3)	161 (93)	13 (7)	
NSMC (n = 4)	515 (8)	13 (3)	502 (97)	

Abbreviations: BWH, Brigham & Women's Hospital; MFM, Maternal Fetal Medicine; MGH, Massachusetts General Hospital; NP, nurse practitioner; NSMC, North Shore Medical Center; NWH, Newton-Wellesley Hospital; OB/Gyn, obstetrics and gynecology; PA, physician’s assistant; REI, reproductive endocrinology and infertility.

^a^ Missing: comorbidities (n = 20); insurance (n = 76).

^b^ The number of ordering clinicians for each specialty is denoted by *n.*

^c^ Other medical specialties includes 3 clinical geneticists (ordered 43 tests), 7 internal medicine/family medicine physicians (ordered 12 tests), and 1 pulmonologist (ordered 1 test).

^d^ The number of clinic locations affiliated with each hospital in the analysis is denoted by *n.*

### Multilevel Factors Associated With Preconception Carrier Screening

Table 2 provides the estimates from the multilevel logistic regression model. In model 1 that includes only fixed effects of the hospital affiliation and random effects of the ordering clinician- and clinic-level effects, we find that patients screened at MGH and NSMC hospital-affiliated sites were less likely to receive preconception screening compared to those receiving care at BWH-affiliated sites. The ICC for clinician-level effects was estimated to be 0.49 (49% of the variability in timing was associated with clinician-level effects), and the ICC for clinic-level effects was estimated to be 0.28 (28% was associated with clinic-level effects); and the corresponding MOR were 12.64 (clinician level) and 6.86 (clinic level), which were comparable in magnitude to the hospital affiliation fixed effects. These results demonstrate high variability in carrier screening timing between clinicians and clinics. For example, the MOR indicates that patients within the same hospital receiving care from randomly sampled pairs of clinicians and clinics would have a median of more than 12 and 6 times the odds between different clinicians and clinics.

**Table 2.** Fixed Effects Estimates and Measures of Residual Variability From Multilevel Logistic Regression Models

Factors	Model 1 (null model)^a^		Model 2 (specialty)^a^		Model 3 (patient)^a^		Model 4 (full model)^a^	
	OR (95% CI)	*P* value	OR (95% CI)	*P* value	OR (95% CI)	*P* value	OR (95% CI)	*P* value
Intercept	1.61 (0.49-5.33)	.43	0.46 (0.16 to 1.27)	.13	1.27 (0.36-4.53)	.71	0.35 (0.11-1.11)	.07
Age, y								
18-25	NA	NA	NA	NA	1 [Reference]		1 [Reference]	
26-30	NA	NA	NA	NA	1.07 (0.65-1.76)	.79	1.01 (0.59-1.71)	.99
31-35	NA	NA	NA	NA	0.88 (0.54-1.44)	.62	0.82 (0.49-1.38)	.46
36-40	NA	NA	NA	NA	1.01 (0.6-1.68)	.98	0.95 (0.55-1.64)	.86
≥41	NA	NA	NA	NA	1.16 (0.57-2.37)	.69	1.03 (0.48-2.2)	.95
Race and ethnicity								
Asian	NA	NA	NA	NA	0.79 (0.56-1.1)	.16	0.79 (0.55-1.13)	.19
Black non-Hispanic	NA	NA	NA	NA	0.59 (0.39-0.88)	.009	0.58 (0.38-0.89)	.01
Hispanic	NA	NA	NA	NA	0.6 (0.36-1.02)	.06	0.63 (0.36-1.09)	.10
White non-Hispanic	NA	NA	NA	NA	1 [Reference]		1 [Reference]	
Other/multiple	NA	NA	NA	NA	0.38 (0.26- to 0.57)	<.001	0.38 (0.25- to 0.58)	<.001
Unknown/missing	NA	NA	NA	NA	1.16 (0.23-5.81)	.86	1.35 (0.26-7.04)	.73
Insurance								
Public	NA	NA	NA	NA	1 [Reference]		1 [Reference]	
Private	NA	NA	NA	NA	1.83 (1.31 to 2.57)	<.001	1.85 (1.29 to 2.66)	.001
None	NA		NA	NA	2.36 (1.6-3.48)	<.001	2.39 (1.58-3.61)	<.001
Comorbidity								
0 comorbidity	NA	NA	NA	NA	1 [Reference]		1 [Reference]	
1 comorbidity	NA	NA	NA	NA	0.75 (0.57-1)	.05	0.75 (0.56 to 1.01)	.06
2 or more	NA	NA	NA	NA	2.04 (1.15-3.63)	.02	1.87 (1.01-3.44)	.05
Clinician specialty								
OB/Gyn	NA	NA	(Reference)				1 [Reference]	
REI	NA	NA	307.82 (90.39-1048.24	<.001	NA	NA	419.56 (117.39-1499.59)	<.001
MFM	NA	NA	1.8 (0.73-4.43	.2	NA	NA	1.87 (0.75-4.64)	.18
Midwife	NA	NA	0.09 (0.02-0.34	<.001	NA	NA	0.14 (0.03-0.55)	.005
PA/NP	NA	NA	0.72 (0.2-2.53	.60	NA	NA	0.58 (0.16-2.12)	.41
Other	NA	NA	1 186 911 461 (0 to NA)	.26	NA	NA	1 731 213 311 (0 to NA)	.54
Hospital affiliation								
BWH	1 [Reference]		1 [Reference]		1 [Reference]		1 [Reference]	
MGH	0.08 (0.01-0.52)	.008	0.12 (0.03-0.59	.01	0.05 (0.01-0.32)	.002	0.08 (0.02-0.39)	.002
NWH	0.31 (0-22.59)	.59	0.12 (0-3.61)	.02	0.19 (0-11.99)	.44	0.07 (0-2.01)	.12
NSMC	0.01 (0-0.24)	.004	0.08 (0.01-0.77)	.03	0.01 (0-0.2)	.002	0.06 (0.01-0.63)	.02

Abbreviations: BWH, Brigham & Women's Hospital; MGH, Massachusetts General Hospital; NSMC, North Shore Medical Center; NWH, Newton Wellesley Hospital; MFM, Maternal Fetal Medicine; NA, not applicable; NP, nurse practitioner; OB/Gyn, obstetrics and gynecology; PA, physician's assistant; REI, reproductive endocrinology and infertility.

^a^ Model 1 contains fixed effect for hospital affiliate and random intercepts for distinct clinicians and clinics; model 2 = model 1 + fixed effects for clinician specialty; model 3 = model 1 + fixed effects for patient-level characteristics; model 4 (full) = model 1 + fixed effects for clincian specialty and patient-level characteristics.

Model 2 and model 3 additionally adjusts for clinician specialty and patient-level characteristics, respectively. These models help quantify the amount of variation explained by the addition of clinician- and patient-level fixed effects relative to model 1. Adjusting for clinician specialty explained greater variability (model 2) than adjusting for patient-characteristics (model 3), as evidenced by the decrease in the variance components of the random effects (PCV = 0.64), which corresponds also to lower SDs for the random effects, ICCs, and MORs when going from model 1 to model 2. However, substantial variability remained unexplained after accounting for specialty. The clinician-level ICC of 0.31 indicated that 31% of residual variation on the log-odds scale remained unexplained across clinicians. This corresponds to a MOR of 4.55, which remains large (Table 3). In contrast, adjusting for patient-level characteristics through model 3 led to minimal changes in these measures of variability in the contextual effects.

**Table 3.** Measures of Residual Variability for Random Effects

Factors	Model 1 (null model)^a^	Model 2 (specialty)^a^	Model 3 (patient)^a^	Model 4 (full model)^a^
Clinician-level				
Standard deviation	2.66	1.59	2.71	1.59
Proportional change in variance	[Reference]	0.64	–0.04	0.64
Intraclass correlation coefficient	0.49	0.31	0.51	0.31
Median odds ratio	12.64	4.55	13.26	4.55
Clinic-level				
Standard deviation	2.02	1.56	1.92	1.5
Proportional change in variance	[Reference]	0.4	0.09	0.45
Intraclass correlation coefficient	0.28	0.3	0.26	0.28
Median odds ratio	6.87	4.44	6.27	4.19

^a^ Model 1 contains fixed effect for hospital affiliate and random intercepts for distinct clinicians and clinics; model 2 = model 1 + fixed effects for clinician specialty; model 3 = model 1 + fixed effects for patient-level characteristics; model 4 (full) = model 1 + fixed effects for clinician specialty and patient-level characteristics.

In the fully adjusted model (model 4), measures of contextual effects are similar to those for model 2. This indicates that adjusting for patient-level factors beyond specialty in the model did not explain much clinician- or clinic-level residual variation, whereas adding specialty to the model adjusting for patient-level factors explained a substantial amount of clinician-level variation. In terms of the fixed effects, we find that hospital affiliation, clinician specialty, and certain individual patient characteristics are significantly associated with screening timing. Patients receiving care from a REI specialist were significantly more likely to have preconception screening relative to those receiving care from general obstetrician-gynecologists (OR, 419.56; 95% CI, 117.39-1499.59), whereas patients receiving care from midwives were less likely to have preconception screens compared with obstetrician-gynecologists (OR, 0.14; 95% CI, 0.03-0.55). At the patient level, non-Hispanic Black patients (OR, 0.58; 95% CI, 0.38-0.89) and patients with other or multiple races and ethnicities (OR, 0.38; 95% CI, 0.25-0.58) were less likely to receive preconception testing compared with White patients. Those with private insurance (OR, 1.85; 95% CI 1.29-2.66) or no payor listed (OR, 2.39; 95% CI, 1.58-3.61) were more likely to receive preconception screening compared with those with public insurance. Those with at least 2 comorbidities were more likely to receive preconception screening compared with those with no comorbidities (OR, 1.87; 95% CI, 1.01-3.44).

### Heterogeneity in Timing of Carrier Screening by Clinician and Clinic

The predicted probability of preconception screening was highly heterogeneous across clinicians by hospital affiliation (Figure). A greater proportion of clinicians were estimated to have moderate-to-high probability of performing preconception testing at BWH-hospital affiliated sites than at MGH-, NWH-, and NSMC-affiliated sites. Probabilities of performing preconception screening were also heterogenous across clinics and, in some cases, within clinics with multiple clinician types (eFigure 2 in the Supplement). REI and other medical specialists consistently exhibited high probabilities of performing preconception screens, midwife clinicians had low probabilities, and there was variation among OB/Gyn and MFM clinicians that varied by hospital affiliation and clinical site.

**Figure.**
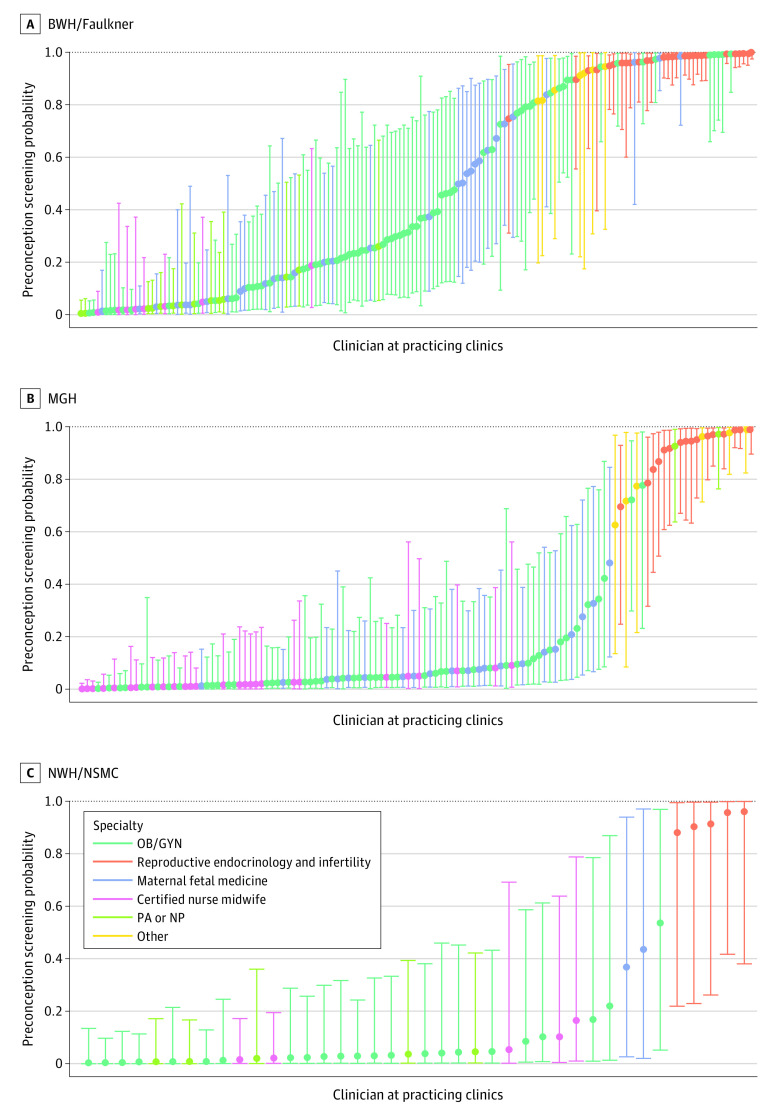
Predicted Probability of Preconception Screening Across Clinicians Based on Model 1 by Site BWH indicates Brigham & Women’s Hospital; MGH, Massachusetts General Hospital; NP, nurse practitioner; NSMC, North Shore Medical Center; NWH, Newton Wellesley Hospital; OB/GYN, obstetrics and gynecology; PA, physician's assistant.

### Explanatory Interviews

Only 9 of the 47 clinicians contacted participated in our interviews, including 3 REI physicians, 3 MFM physicians, 2 General OB/Gyn physicians, and one PA (eTable 4 in the Supplement). Clinicians generally supported performing carrier screening prior to pregnancy, but noted several perceived barriers to implementation at the 1) patient level, such as perception of interference with a natural process, perceived value of testing, cost considerations, engaging patients with lower educational level or language barriers, 2) clinician-level, including uncertainty about best practices for offering preconception screening and needing to engage clinicians who see females of reproductive age prior to pregnancy, and 3) systems-levels, such as the need to develop and implement simplified processes to facilitate test ordering and follow-up (Table 4).

**Table 4.** Explanatory Interview Results Organized by CFIR Domain

Clinicians’ knowledge and beliefs about preconception carrier screening	Representative quotation
Clinicians overwhelmingly endorsed support for preconception screening. Areas of uncertainty about how to screen include when to screen, what conditions to test for, and how to facilitate partner testing.	“I guess if it’s done too far prior to a pregnancy, patients may not know that they had it done. Patients may forget about the results. It may be out of date.…So, if someone had carrier screening 10 years ago, it could be that we recommend repeating it anyway.” – MFM
**Relative advantages of preconception carrier screening compared with prenatal screening**	
More time for partner testing. More time for genetic counseling. Greater reproductive options, such as the ability to pursue preimplantation embryo screening. The potential to reduce birth defects/detect rare diseases. Reduced stress for future parent(s).	“I think when people are already pregnant and you tell them potentially bad news or you reveal an uncertainty, the anxiety is much higher, because they feel that every day is ticking down. They feel this rush to make a decision, or they start panicking….It’s less stressful to learn all that before you have an actual pregnancy.” – PA “The majority of my practice are couples seeking fertility, or single women....In my circumstance, we work very, very hard to help people who are not easily getting pregnant…and it would be really pretty unfortunate if we had an opportunity to screen for a condition, even though it's rare, and did not do it.” – REI
**Relative disadvantages of preconception screening compared with prenatal screening**	
Patient costs, partner testing costs, and trouble with insurance coverage. The difficulty of conveying the value of testing prior to pregnancy. Uncertainty about best practices when reproductive partner is unknown. Lost or forgotten results Perceived interference with a natural reproductive process.	“…Unfortunately, their insurance is not going to cover it, therefore they have to prepare for likely a…few hundred dollars, compared to something that seems much more…relevant to an immediate pregnancy.” – PA “I think some people are hesitant to know too much information and sort of feel like we’re interfering in a process that should be more natural.” – REI “Like getting your blood drawn? I can’t think of any [disadvantages].” – OB/Gyn
**Areas for process improvement and/or barriers to implementation**	
Simplifying test-related processes including pretest counseling, ordering, result follow-up, and partner testing coordination. Engaging non-OB/Gyn clinicians to reach target population for preconception screening. Considering alternative models for screening that did not depend on the clinician as a gatekeeper to screening. Constraints on time and competing priorities were also described as barriers to screening.	“Who is seeing reproductive age women for routine care, and that would be the place to deploy offering this type of testing. So primary care physicians, general OB/Gyns...” – MFM “They could just pay, I think it might be over $100, and get it done on their own through these outside companies.” – OB/Gyn “So the typical person…with whom I can really engage in this is someone who isn’t on a lot of medication, doesn’t have an extensive psychiatric history…all those other problems, those really take precedence...” – OB/Gyn “I know that some of the primary care clinics here at the Brigham are trying to do it as a preconception screening tool, which I think is a great step.” - MFM
**Patient needs and resources**	
High-quality pretest counseling and patient education such as direct clinical counseling, informational videos, and genetic counseling referrals. Tools to overcome cultural and language barriers.	“I usually end the appointment saying, ‘By the way, this is a test we can offer during the pregnancy, but actually we can do it before the pregnancy, and it’s more valuable then,’…And…most people say no.…I think the barrier is this idea that somehow it’s not relevant until I’m pregnant.” – PA

Abbreviations: CFIR, Consolidated Framework for Implementation Research; MFM, Maternal Fetal Medicine Specialist; OB/Gyn, general obstetrician and gynecologist; PA, physician’s assistant; REI, reproductive endocrinology and infertility specialist.

## Discussion

We found that 37% of patients in our cohort (2438 of 6509) had preconception screening, despite clinical guidelines supporting preconception screening as preferable to prenatal screening.^8^ We observed that site of care, clinician specialty, and patient race, insurance payor, and comorbidity status were associated with carrier screening timing. We also quantified residual variability at the clinician and clinic levels after accounting for specialty.

It is not surprising that a minority of patients received preconception carrier screening, and that most of these patients were being cared for by specialists such as REI who routinely engage patients prior to conception. However, in the US just under half of pregnancies were unintended in 2011 (45%)^29^ so engaging clinicians who interact routinely with patients outside of reproductive planning, such as internists and family practice clinicians,^30^ could be critical to improving access to preconception screening. In our data, it was interesting to note both that the 7 internal and family medicine physicians who ordered screening did so exclusively in the preconception period, even though they only accounted for a very small proportion of screens. Our small group of interviewed clinicians also suggested engaging clinicians that may see patients prior to pregnancy. This strategy has been supported elsewhere.^31^

Certain patient characteristics were associated with variability in carrier screening timing after adjustment. Patients with multiple co-morbidities were more likely to be screened preconception, perhaps due to more contact with the health care system. Lower odds of preconception testing among Blacks, other race/ethnicities, and publicly-insured patients raise concerns about disparities in access to preconception screening even after adjustment, which were echoed in our clinician interviews and by others.^32^ Expanding population-based preconception screening programs could amplify these disparities if not done thoughtfully, given that Black non-Hispanic patients have had higher rates of unplanned pregnancies,^29^ and that 42% of pregnancies in the US are paid for by Medicaid,^33^ which is a much higher proportion than the 21% of patients in our cohort who had public insurance as the primary payor. Designing inclusive screening programs, implementing policies that would allow equal access to testing despite insurance product,^34^ and ensuring carrier screens are beneficial to diverse genetic ancestry groups,^9^ are potential considerations.

Although the quantitative results demonstrated a predominance of prenatal carrier screening across MGB, our limited sample of interviewed clinicians overwhelmingly expressed support for preconception screening. Interviewees also expressed substantial uncertainty about the best practices for implementing population-based preconception screening offerings. Fortunately, single-group^35^ and randomized clinical trials^36^ are evaluating the impact of preconception carrier screening on patient, clinician, and health care system-level outcomes, which could inform best practices.

### Limitations

This study had some limitations. Our study was limited to patients who completed testing ordered at a MGB clinical site through a single laboratory, rather than the population eligible for testing, given difficulty capturing this larger group due to variability in timing of preconception screening and concerns about reliably capturing external testing. We acknowledge that patients completing carrier screening are likely to differ from the general population and may have fewer socioeconomic barriers to clinical care, limiting generalizability. Future work could seek to understand barriers to preconception screening among those who either were not offered or did not complete testing, to further understand barriers to preconception screening.

We also observed unexplained residual variability after adjustment, suggesting a need to identify other factors contributing to variability of carrier screening timing. Disappointingly, only a small minority of clinicians approached participated in our interviews. Despite this, the interviews help us to hypothesize about unmeasured factors that could contribute to variation, such patient willingness to undergo screening, competing clinical needs, and the presence or absence of operational supports, such as the involvement of clinical team members like nurses and genetic counselors who were not captured in this study, given they cannot order genetic testing.

## Conclusions

This analysis provides unique insights into multilevel factors associated with carrier screening timing. Given clinician specialty was the strongest measured factor of carrier screening timing, engaging clinicians interacting with non-pregnant females of reproductive age could be a place to start to increase access to preconception screening.

## Notes

Supplement. **eAppendix.** Supplementary Materials **eFigure 1.** Cohort Creation **eTable 1.** Comparing Characteristics of Patients by Data Types Available **eFigure 2.** Predicted Probability of Pre-Conception Screening Across Providers Based on Model 1 by Site-Clinic **eFigure 3.** Number of Conditions Tested for on Carrier Screening Panel by Timing and Provider Specialty **eTable 3.** Frequencies of Positive and Negative Carrier Screen Results by Timing of Carrier Screening **eTable 4.** Characteristics of Ordering Providers Who Participated in Interviews (N=9)
