# Supplementary material for: Association of Patient and Site-of-Care Characteristics With Reproductive Carrier Screening Timing in a Large Integrated Health System
Source: JAMA Netw Open. 2022 Nov 8;5(11):e2240829. doi: 10.1001/jamanetworkopen.2022.40829 (PMC9644263; doi:10.1001/jamanetworkopen.2022.40829)
Supplement: Supplement. — eAppendix. Supplementary Materials eFigure 1. Cohort Creation eTable 1. Comparing Characteristics of Patients by Data Types Available eFigure 2. Predicted Probability of Pre-Conception Screening Across Providers Based on Model 1 by Site-Clinic eFigure 3. Number of Conditions Tested for on Carrier Screening Panel by Timing and Provider Specialty eTable 3. Frequencies of Positive and Negative Carrier Screen Results by Timing of Carrier Screening eTable 4. Characteristics of Ordering Providers Who Participated in Interviews (N=9) [file jamanetwopen-e2240829-s001.pdf]

Supplemental Online Content

Hull LE, Cheng D, Hallman MH, Rieu-Werden ML, Haas JS. Association of patient and site-of-care characteristics with reproductive carrier screening timing in a large integrated health system. *JAMA Netw Open.* 2022;5(11):e2240829. doi:10.1001/jamanetworkopen.2022.40829

eAppendix. Supplementary Materials

eFigure 1. Cohort Creation

eTable 1. Comparing Characteristics of Patients by Data Types Available

eFigure 2. Predicted Probability of Pre-Conception Screening Across Providers Based on Model 1 by Site-Clinic

eFigure 3. Number of Conditions Tested for on Carrier Screening Panel by Timing and Provider Specialty

eTable 3. Frequencies of Positive and Negative Carrier Screen Results by Timing of Carrier Screening

eTable 4. Characteristics of Ordering Providers Who Participated in Interviews (N=9)

This supplemental material has been provided by the authors to give readers additional information about their work.

© 2022 Hull LE et al. *JAMA Network Open.*

eAppendix. Supplementary Materials

Supplementary Qualitative Methods for Explanatory Interviews

Explanatory Interviews: Overview

To better understand variability in carrier screening, we conducted semi-structured telephone interviews of ordering providers.

Recruitment

Providers were recruited directly via email between 9/22/20 and 1/15/2021. We purposively focused on outreach to providers who had either ordered little to no preconception screening in FY2019, ordered both prenatal and preconception screens in the same year, or ordered carrier screens testing for different numbers of recessive conditions to identify providers who could speak to variability in practice, who could serve as cases helping to explain and explore the observed variability in our quantitative data. 47 invitations were sent; 9 providers agreed to participate. Reasons for non-participation were not ascertained. No remuneration was provided. Providers received information about the study as part of email recruitment and during the verbal consent process using procedures approved by the MGB Institutional Review Board.

Interview Guide Development

We used the Consolidated Framework for Implementation Research (CFIR),[28] an implementation science framework used widely to elucidate the barriers to implementation of evidence-based healthcare pratices,[29] to direct interview guide design. The full copy of the interview guide is available in this supplement. Given our interest in providers' beliefs about preconception carrier screening, questions were developed using the assistance of the CFIR interview guide tool (https://cfirguide.org/guide/app/#/), with a specific focus on developing questions related to the following Constructs: Intervention Characteristics, Characteristics of Individuals, and Outer Setting. The interview guide was piloted amongst the research team, as well as a group of volunteer genetic counselors, prior to use.

Data Collection

© 2022 Hull LE et al. *JAMA Network Open.*

Phone interviews were scheduled for 30 minutes and were conducted either from the investigator's private home office or research office. They were conducted by LEH, a junior faculty physician-investigator with training in qualitative methods. MH was present on the phone interviews and took field notes. A transcription service (Transcribe Me, https://www.transcribeme.com/) was used to transcribe telephone interview transcripts. Transcripts were reviewed to ensure no personal identifiers were contained. If so they were removed. Field notes and the original recordings were consulted as needed if there were gaps in transcription during the cleaning process (performed by MH).

Data Analysis

A rapid qualitative matrix approach was used for data analysis.[30,31] First, the transcripts were broken up by question and the responses were organized into cells in Microsoft Excel notebooks based upon the CFIR construct each question was designed to address. LEH and MHH independently reviewed and coded each cell and then met to compare codes by CFIR construct, reconcile any differences, and discuss resulting themes. Results were organized for presentation by CFIR domain.

© 2022 Hull LE et al. *JAMA Network Open.*

STROBE Checklist

STROBE Statement—Checklist of items that should be included in reports of ***cross-sectional studies***

	Item No	Recommendation	Page No
**Title and abstract**	1	(*a*) Indicate the study's design with a commonly used term in the title or the abstract	3
		(*b*) Provide in the abstract an informative and balanced summary of what was done and what was found	3-4
**Introduction**			
Background/rationale	2	Explain the scientific background and rationale for the investigation being reported	5
Objectives	3	State specific objectives, including any prespecified hypotheses	5-6
**Methods**			
Study design	4	Present key elements of study design early in the paper	6
Setting	5	Describe the setting, locations, and relevant dates, including periods of recruitment, exposure, follow-up, and data collection	6-8
Participants	6	(*a*) Give the eligibility criteria, and the sources and methods of selection of participants	6-7
Variables	7	Clearly define all outcomes, exposures, predictors, potential confounders, and effect modifiers. Give diagnostic criteria, if applicable	7-8
Data sources/ measurement	8*	For each variable of interest, give sources of data and details of methods of assessment (measurement). Describe comparability of assessment methods if there is more than one group	*6-8*
Bias	9	Describe any efforts to address potential sources of bias	6-8, Supplement
Study size	10	Explain how the study size was arrived at	6-8, SFig 1
Quantitative variables	11	Explain how quantitative variables were handled in the analyses. If applicable, describe which groupings were chosen and why	7-8
Statistical methods	12	(*a*) Describe all statistical methods, including those used to control for confounding	8-9
		(*b*) Describe any methods used to examine subgroups and interactions	n/a
		(*c*) Explain how missing data were addressed	8-9, Supplement
		(*d*) If applicable, describe analytical methods taking account of sampling strategy	8-9
		(*e*) Describe any sensitivity analyses	n/a
**Results**			
Participants	13*	(a) Report numbers of individuals at each stage of study—eg numbers potentially eligible, examined for eligibility, confirmed eligible, included in the study, completing follow-up, and analysed	SFig 1, 10
		(b) Give reasons for non-participation at each stage	SFig1, Stable 1
		(c) Consider use of a flow diagram	SFig 1
Descriptive data	14*	(a) Give characteristics of study participants (eg demographic, clinical, social) and information on exposures and potential confounders	Table 1
		(b) Indicate number of participants with missing data for each variable of interest	Table 1
Outcome data	15*	Report numbers of outcome events or summary measures	Table 1
Main results	16	(*a*) Give unadjusted estimates and, if applicable, confounder-adjusted estimates and their precision (eg, 95% confidence interval). Make clear which confounders were adjusted for and why they were included	Table 2
		(*b*) Report category boundaries when continuous variables were categorized	Table 2
		(*c*) If relevant, consider translating estimates of relative risk into absolute risk for a meaningful time period	n/a

© 2022 Hull LE et al. *JAMA Network Open.*

Other analyses	17	Report other analyses done—eg analyses of subgroups and interactions, and sensitivity analyses	n/a
**Discussion**			
Key results	18	Summarise key results with reference to study objectives	13
Limitations	19	Discuss limitations of the study, taking into account sources of potential bias or imprecision. Discuss both direction and magnitude of any potential bias	14-15
Interpretation	20	Give a cautious overall interpretation of results considering objectives, limitations, multiplicity of analyses, results from similar studies, and other relevant evidence	14-15
Generalisability	21	Discuss the generalisability (external validity) of the study results	14-15
**Other information**			
Funding	22	Give the source of funding and the role of the funders for the present study and, if applicable, for the original study on which the present article is based	15

© 2022 Hull LE et al. *JAMA Network Open.*

COREQ Checklist

COREQ (COnsolidated criteria for REporting Qualitative research) Checklist

A checklist of items that should be included in reports of qualitative research. You must report the page number in your manuscript where you consider each of the items listed in this checklist. If you have not included this information, either revise your manuscript accordingly before submitting or note N/A.

Topic	Item No.	Guide Questions/Description	Reported on Page No.
**Domain 1: Research team and reflexivity**			
Personal characteristics			
Interviewer/facilitator	1	Which author/s conducted the interview or focus group?	Supplement
Credentials	2	What were the researcher's credentials? E.g. PhD, MD	Supplement
Occupation	3	What was their occupation at the time of the study?	Supplement
Gender	4	Was the researcher male or female?	Supplement
Experience and training	5	What experience or training did the researcher have?	Supplement
Relationship with participants			
Relationship established	6	Was a relationship established prior to study commencement?	Supplement
Participant knowledge of the interviewer	7	What did the participants know about the researcher? e.g. personal goals, reasons for doing the research	Supplement
Interviewer characteristics	8	What characteristics were reported about the inter viewer/facilitator? e.g. Bias, assumptions, reasons and interests in the research topic	Supplement
**Domain 2: Study design**			
Theoretical framework			
Methodological orientation and Theory	9	What methodological orientation was stated to underpin the study? e.g. grounded theory, discourse analysis, ethnography, phenomenology, content analysis	Supplement
Participant selection			
Sampling	10	How were participants selected? e.g. purposive, convenience, consecutive, snowball	Supplement
Method of approach	11	How were participants approached? e.g. face-to-face, telephone, mail, email	Supplement
Sample size	12	How many participants were in the study?	Methods/Supplement
Non-participation	13	How many people refused to participate or dropped out? Reasons?	Methods/Supplement
Setting			
Setting of data collection	14	Where was the data collected? e.g. home, clinic, workplace	Supplement
Presence of non-participants	15	Was anyone else present besides the participants and researchers?	Supplement
Description of sample	16	What are the important characteristics of the sample? e.g. demographic data, date	Supplement
Data collection			
Interview guide	17	Were questions, prompts, guides provided by the authors? Was it pilot tested?	Supplement
Repeat interviews	18	Were repeat inter views carried out? If yes, how many?	Supplement

© 2022 Hull LE et al. *JAMA Network Open.*

Audio/visual recording	19	Did the research use audio or visual recording to collect the data?	Supplement
Field notes	20	Were field notes made during and/or after the inter view or focus group?	Supplement
Duration	21	What was the duration of the inter views or focus group?	Supplement
Data saturation	22	Was data saturation discussed?	Supplement
Transcripts returned	23	Were transcripts returned to participants for comment and/or	Supplement

© 2022 Hull LE et al. *JAMA Network Open.*

Topic	Item No.	Guide Questions/Description	Reported on Page No.
		correction?	
**Domain 3: analysis and findings**			
Data analysis			
Number of data coders	24	How many data coders coded the data?	Supplement
Description of the coding tree	25	Did authors provide a description of the coding tree?	Supplement
Derivation of themes	26	Were themes identified in advance or derived from the data?	Supplement
Software	27	What software, if applicable, was used to manage the data?	Supplement
Participant checking	28	Did participants provide feedback on the findings?	Supplement
Reporting			
Quotations presented	29	Were participant quotations presented to illustrate the themes/findings? Was each quotation identified? e.g. participant number	Supplement
Data and findings consistent	30	Was there consistency between the data presented and the findings?	Supplement
Clarity of major themes	31	Were major themes clearly presented in the findings?	Supplement
Clarity of minor themes	32	Is there a description of diverse cases or discussion of minor themes?	Supplement

Developed from: Tong A, Sainsbury P, Craig J. Consolidated criteria for reporting qualitative research (COREQ): a 32-item checklist for interviews and focus groups. *International Journal for Quality in Health Care*. 2007. Volume 19, Number 6: pp. 349 – 357

Once you have completed this checklist, please save a copy and upload it as part of your submission. DO NOT include this checklist as part of the main manuscript document. It must be uploaded as a separate file.

© 2022 Hull LE et al. *JAMA Network Open*.

Supplemental File 1. Explanatory Interview Guide

Please note that the interview guide below also includes questions about provider perceptions of expanded carrier screening. The analyses presented in this manuscript focused on carrier screening timing and did not include qualitative data related to perceptions of expanded carrier screens.

Section 1. Assessment of understanding of genetic carrier screening and preferences.

**[Interviewer]:** ***Thank you again for taking part in this interview. I am interested in learning more about your perceptions of genetic carrier screening.***	
**Questions**	**CFIR Constructs**
**I'd like to start off by having you describe for me what genetic carrier screening means to you.**<br>• *When is it used?*<br>• *What does it test for?*	Characteristics of Individuals: Knowledge & Beliefs about the Intervention
**Please describe your process for ordering genetic carrier screening. Please take me through what you tell the patient and how you order the test step-by-step.**<br>• *How do you decide when to order carrier screening for a patient?*<br>• *How to you decide what conditions to screen for?*	Individual's current practices – Not from a CFIR Construct
**Please describe your process for sharing the results of carrier screening with your patients.**<br>• *For example, do you or a member of your team let the patient know about the results, and if so, how (e.g. by phone, secure messaging, or other means)?*<br>• *Are there any cases when you wouldn't share the results with your patient(s)?*	Individual's current practices – Not from a CFIR Construct
**Have you heard of expanded carrier screening?**<br>• *If so, could you describe it for me?*	Characteristics of Individuals: Knowledge & Beliefs about the Intervention
**If you could choose when and how to order genetic carrier screening for your patients, what do you think would be your ideal way to offer this type of test?**<br>• *For example, who would order the test, what testing would you order, in what clinical setting would testing be done, and how would the results be followed-up? If you need a minute to think about this, please feel free to do so.*	Perception of best practices for carrier screening – Not from a CFIR construct
**Are you aware of any other clinics, practices, or health care systems that you think are doing a particularly good job providing patients with carrier screening?**	Outer Setting: Peer Pressure

© 2022 Hull LE et al. *JAMA Network Open.*

**How else could you imagine someone using information about their carrier status outside of pregnancy? Take a moment to think about this, if you'd like.**<br>• *Tell me a story about a patient who used their carrier screen information outside of pregnancy….*	Characteristics of Individuals: Knowledge & Beliefs about the Intervention

Section 2. Barriers and facilitators to implementing preconception carrier screening.

**[Interviewer:]** ***Thank you for your responses. We are interested in learning about ways to expand access to preconception carrier screening, or carrier screening performed prior to pregnancy. We'd like to learn more about your perspectives on if and how to expand offering this service to patients, and the barriers that we would need to address to do so.***	
**Questions**	**CFIR Constructs**
**How do you feel about offering preconception carrier screening in your clinical practice?**	Characteristics of Individuals: Knowledge & Beliefs about the Intervention
• *What advantages does preconception carrier screening have compared to prenatal carrier screening?*<br>• *What disadvantages?*	Intervention characteristics: Relative advantage
**What barriers will your patients face to participating in preconception carrier screening?**<br>• *Tell me a story about a barrier you or your patient faced when pursuing preconception carrier screening.*	Outer Setting: Patient Needs & Resources

Section 3. Barriers and facilitators to implementing expanded carrier screening.

**[Interviewer:]** ***We are also interested in barriers you may face to offering expanded carrier screening in your practice, as well as things that can be done to make using expanded carrier screening more accessible. For the purposes of these questions, we will define expanded carrier screening as genetic carrier screening for multiple autosomal and X-linked recessive disorders, beyond a set of core disorders included in current guidelines, that is not based on a patient's self-reported race or ethnicity. The screening tests generally include hundreds of genes. Examples of expanded carrier screens you may have heard of include the Myriad Foresight® Expanded Carrier Screen and the Invitae Comprehensive Carrier Screen, among others.***	
**Questions**	**CFIR Constructs**
**How do you feel about offering expanded carrier screening as the default type of carrier screening performed in your clinical practice?**	Characteristics of Individuals: Knowledge &

© 2022 Hull LE et al. *JAMA Network Open.*

	Beliefs about the Intervention
• *What advantages do expanded carrier screens have compared to more targeted or limited screening?*<br>• *What disadvantages?*	Intervention characteristics: Relative advantage
**What barriers will your patients face to participating expanded carrier screening?**<br>• *Have you heard stories about the experiences of participants with expanded carrier screening? Can you describe a specific story?*	Outer Setting: Patient Needs & Resources

Section 4. Conclusion

[Interviewer:] ***Thank you for answering our questions. Before we finish, is there anything else that you'd like share with me about genetic carrier screening?***

Thank you again for your participation.

© 2022 Hull LE et al. *JAMA Network Open.*

eFigure 1. Cohort Creation

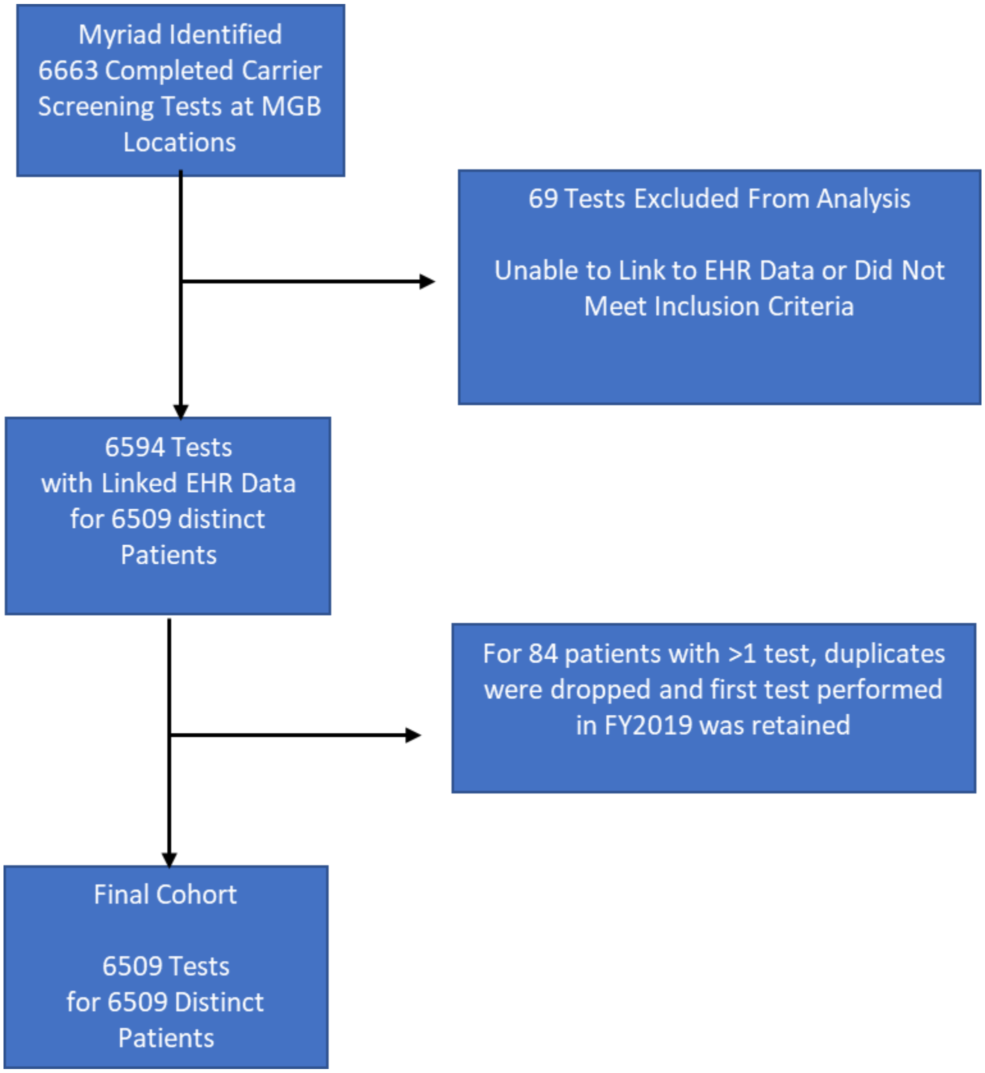

*61 out of the 84 patients with >1 test had the same pregnancy status for both screens

© 2022 Hull LE et al. *JAMA Network Open.*

eTable 1. Comparing Characteristics of Patients by Data Types Available*

		Myriad Data and EHR Order	Myriad Data but No EHR Order Found (EHR Data Available for Linkage)	EHR Order Only but No Myriad Data	p-value**
**Characteristics**	6716	5709 (85%)	800 (12%)	207 (3%)	
Pregnancy Status					*<0.001*
Pregnant	4333	3600	587	146	
Not Pregnant	2383	2109	213	61	
Age Group					*<0.001*
18-25	611	472	125	14	
26-30	1795	1503	240	52	
31-35	2696	2313	298	85	
36-40	1327	1166	117	44	
41 or Greater	287	255	20	12	
Race/Ethnicity					*<0.001*
White NH	4115	3400	586	129	
Black NH	757	664	72	21	
Hispanic	8	1	0	7	
Asian	832	745	64	23	
Other	783	710	53	20	
Unknown	221	189	25	7	
*Insurance Type****					*<0.001*
Public	1436	1235	170	31	
Private	3566	3012	458	96	
None	1712	1462	170	80	
*# Comorbidities****					*<0.001*
No Comorbidities	5440	4637	631	172	
1 Comorbidity	1077	915	134	28	
2 or More Comorbidities	175	156	17	2	

Abbreviations: EHR=electronic health record; NH = Non-Hispanic
*Could not compare cohorts by ordering location, ordering provider ID, clinic location on order requisition, or provider specialty as these data were not available for those with an EHR order only

**p-value based on Person's Chi-square test except in case of low cell counts (<5 in any cell) where Fisher's exact test was used

***Missing: Insurance (2); Number of comorbidities (24)

© 2022 Hull LE et al. *JAMA Network Open.*

eFigure 2: Predicted Probability of Pre-Conception Screening Across Providers Based on Model 1 by Site-Clinic

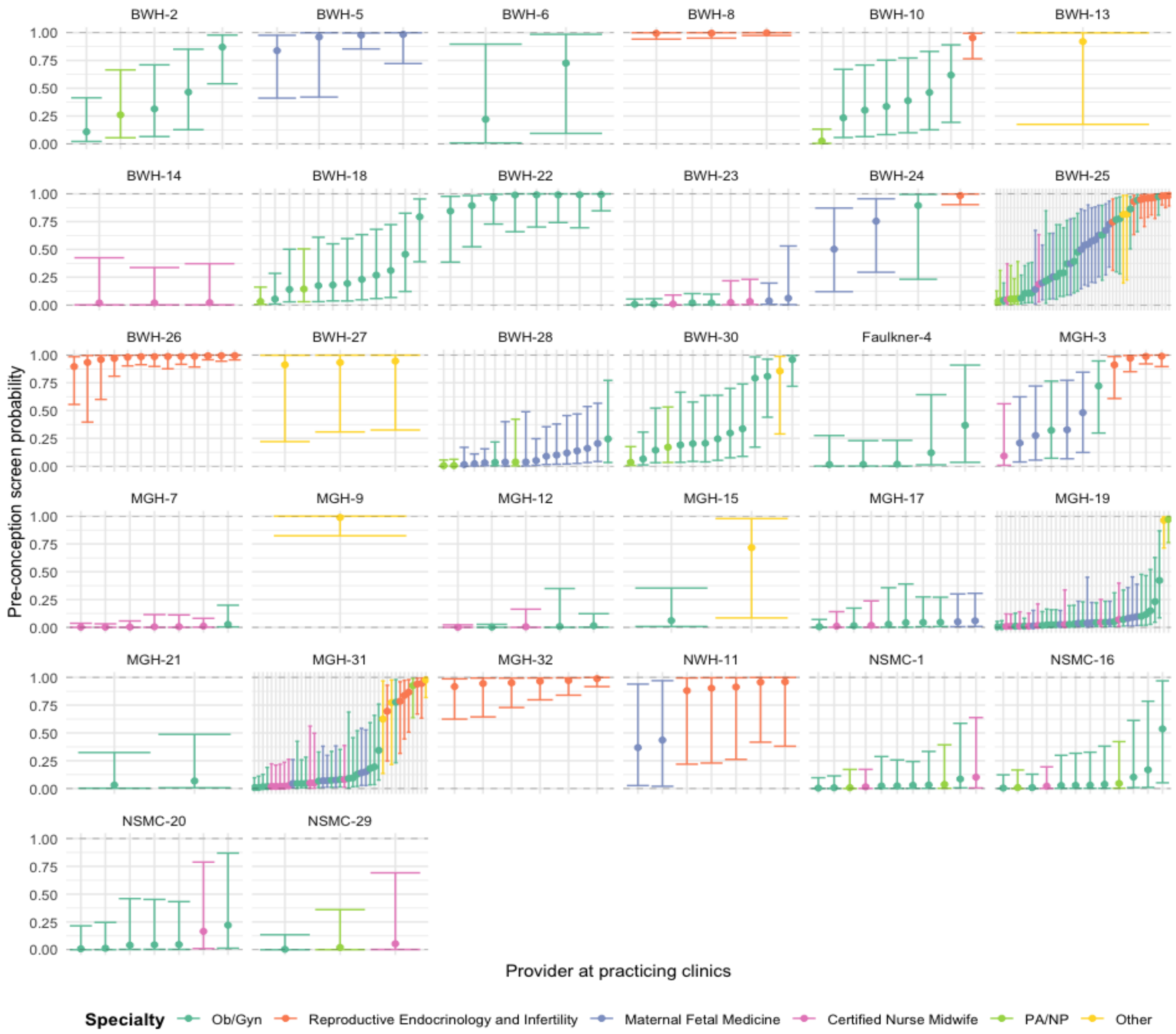

Abbreviations: BWH=Brigham & Women's Hospital; MGH=Massachusetts General Hospital; NWH=Newton Wellesley Hospital; NSMC = North Shore Medical Center; OB/Gyn=Obstetrics & Gynecology; REI=Reproductive Endocrinology and Infertility; MFM=Maternal Fetal Medicine; PA=Physician's Assistant; NP=Nurse Practitioner

© 2022 Hull LE et al. *JAMA Network Open.*

eFigure 3. Number of Conditions Tested for on Carrier Screening Panel By Timing and Provider Specialty

Figure 3A: Number of Conditions Tested for on Carrier Screen Panel by Pregnancy Status

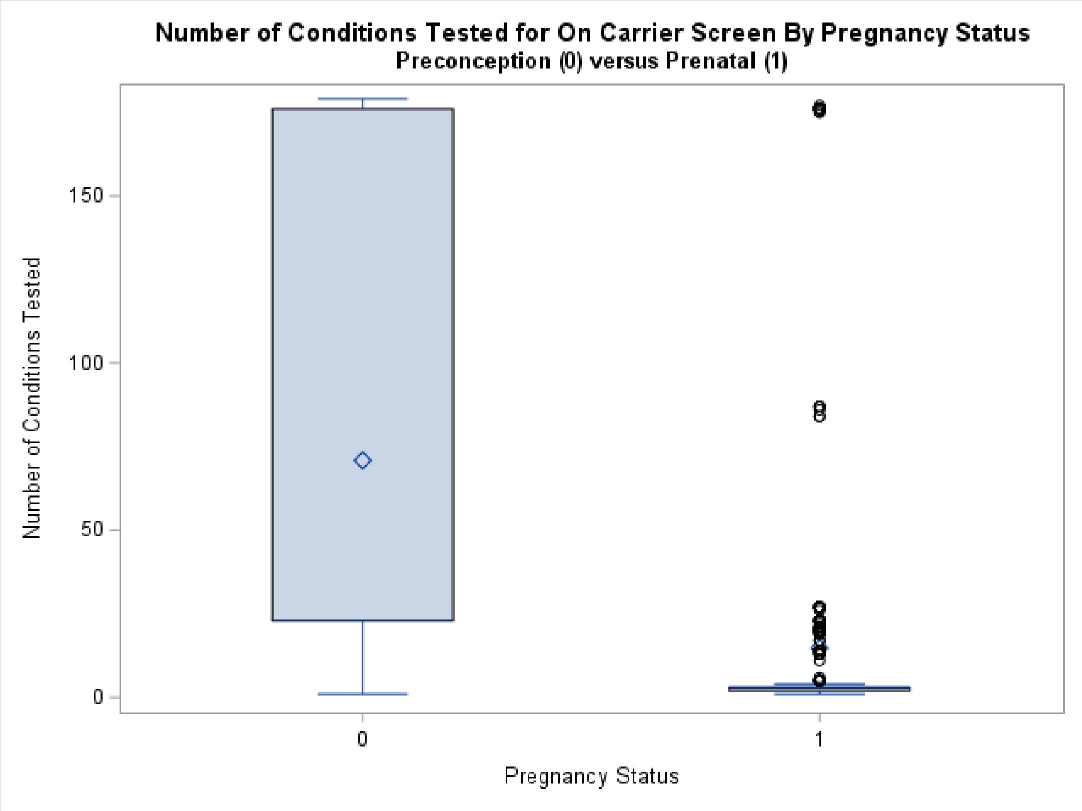

© 2022 Hull LE et al. *JAMA Network Open.*

eFigure 3B: Number of Conditions Tested for on Carrier Screen Panel by Pregnancy Status & Ordering Provider Specialty

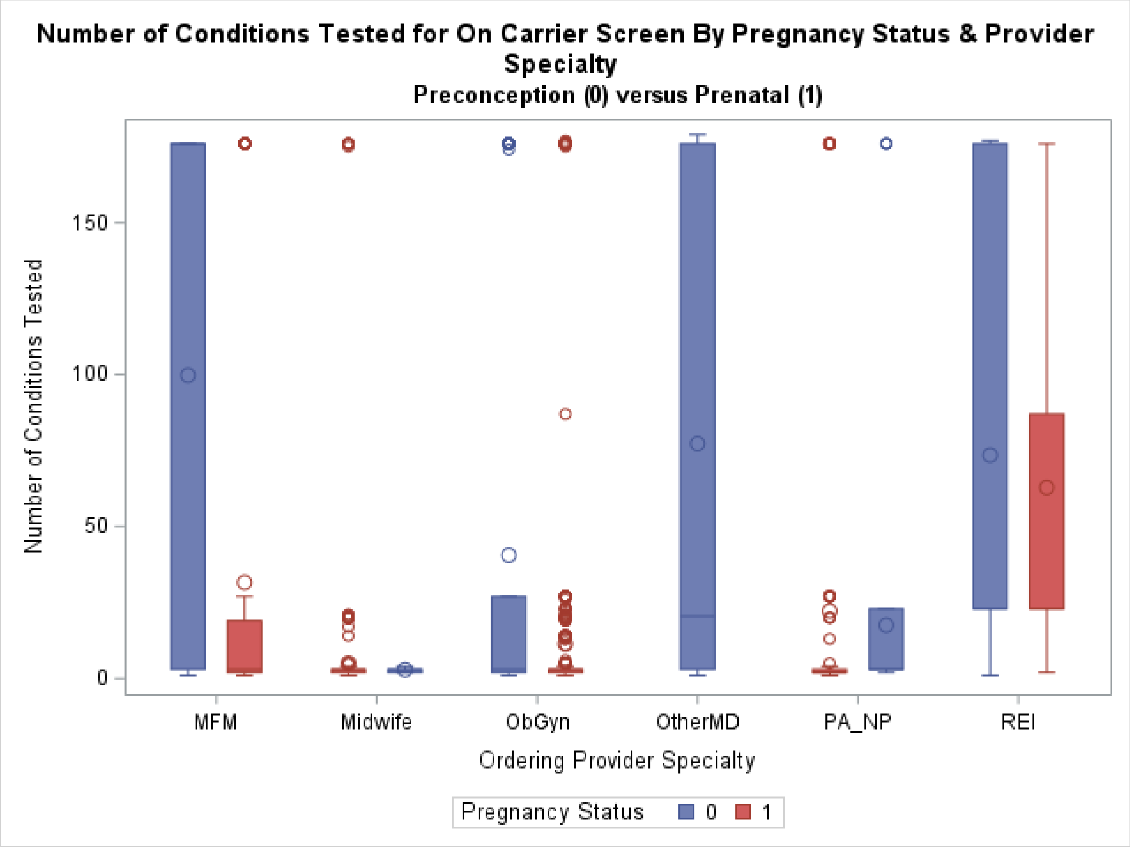

eTable 3. Frequencies of Positive and Negative Carrier Screen Results by Timing of Carrier Screening*

	Prenatal	Preconception
Result		
Positive	478	745
Negative	3593	1693
*These are raw frequencies and have not been adjusted for panel size or carrier frequency by ancestry. Differences in positive and negative results are likely associated with the size of the carrier screening panels in both groups. No formal analysis has been performed at this time.		

© 2022 Hull LE et al. *JAMA Network Open.*

eTable 4. Characteristics of Ordering Providers who Participated in Interviews (N=9)

Provider Specialty	Total Carrier Screens Ordered In FY2019 by Provider	% of Tests Ordered Preconception
REI	108	100%
REI	77	99%
REI	116	91%
MFM	54	69%
MFM	11	45%
OB/Gyn	18	39%
MFM	43	12%
PA-C	144	2%
OB/Gyn	79	0%

© 2022 Hull LE et al. *JAMA Network Open.*
